# A case report of metastatic renal cell carcinoma and ANCA associated vasculitis

**DOI:** 10.1186/s12882-025-04247-4

**Published:** 2025-07-03

**Authors:** Maddison Taylor, Cassandra Rawlings, George Kan, Andrew J. Mallett, Michelle Harfield

**Affiliations:** 1 Department of Nephrology, Townsville University Hospital, 100 Angus Smith Drive, Douglas, Townsville, Queensland 4814 Australia; 2 College of Medicine and Dentistry, James Cook University, Townsville, Queensland Australia; 3 Institute for Molecular Bioscience, The University of Queensland, Brisbane, Queensland Australia

**Keywords:** Renal cell cancer, ANCA associated vasculitis, Immunotherapy, Tyrosine kinase inhibitor, Case report

## Abstract

Renal Cell Carcinoma (RCC) may uncommonly present concurrently with anti-neutrophil cytoplasmic antibody (ANCA) associated vasculitis (AAV). Most instances are malignancies discovered incidentally during the work up after a diagnosis of AAV; however, the significant overlap between these disorders potentially suggests a more complex pathophysiology. We present the case of a 53-year-old gentleman who was diagnosed with metastatic RCC who later developed rapidly progressive kidney failure and a vasculitic rash after commencement of a tyrosine kinase inhibitor and was ultimately diagnosed with subsequent concurrent AAV. Treatment included suspending immunotherapy, glucocorticoids, and rituximab induction. This case highlights the unique scenario of concurrent RCC and AAV, including the potential pathophysiology of a pro-inflammatory milieu created in the setting of RCC which permits AAV. It also outlines the complexity of treating an autoimmune disorder in the setting of an active cancer requiring immunotherapy and the difficult balance between these two treatment paradigms.

## Background

ANCA associated vasculitis (AAV) is a pauci-immune systemic, necrotising small vessel vasculitis with a world-wide incidence of 300–421 cases per million persons [1, 2]. There are three main clinical sub-groups of AAV based on their clinical, histological and immunological findings including granulomatosis with polyangiitis (GPA), microscopic polyangiitis and eosinophilic granulomatosis with polyangiitis [3]. The definition of the disease has evolved over the years with the 2022 ACR (American College of Rheumatology) and EULAR (European Alliance of Associations for Rheumatology) developing scoring systems to better characterize AAV disease, in particular GPA [4]. Clinically, AAV can present with non-specific systemic features such as weight loss, malaise and arthralgia. It can also have an index presentation with organ threatening vasculitis including rapidly progressive glomerulonephritis, pulmonary haemorrhage, peripheral nerve involvement and nasal disease [5].

The pathophysiology involves loss of tolerance against neutrophil granule components and the development of ANCA auto-antibodies (anti-neutrophil cytoplasmic antibodies), which subsequently lead to microvascular inflammation, tissue necrosis and inflammatory cell infiltration to blood vessel walls [1]. Essential to the pathogenesis in AAV are the auto-antibodies to myeloperoxidase (MPO) and proteinase 3 (PR-3); although there are rare cases of alternative auto-antigens and even ANCA-negative AAV [1, 6]. Binding of the ANCA auto-antibodies leads to the activation of neutrophils, and propagation of microvascular inflammation [1, 6]. Testing for the presence of ANCA in serum is an essential part of the work up for a glomerulonephritis presentation. This involves testing serum via immunofluorescence for c-ANCA (cytoplasmic pattern) associated with PR3 positivity and p-ANCA (peri-nuclear pattern) consistent with MPO positivity most often [2]. Indirect immunofluorescence must then be followed by the more sensitive and specific ELISA immunoassay for MPO and PR3 positivity; indeed there is a move to transition predominantly to immunoassay for initial investigative work up for AAV [7].

In Australia, the incidence of Renal Cell Carcinoma (RCC) is 13 cases per 100,000 with clear cell RCC being the predominant histological diagnosis [8]. A study in 2015 showed that of those patients presenting with metastatic RCC, 77% received first line therapy with sunitinib and had a median survival of 27.6 months [9]. A minority of cases progress to second line therapy due to side effects, disease progression or other reasons.

There are infrequent reports in the literature of RCC being incidentally or concurrently diagnosed during work up for patients who present with AAV [10]. It is unclear whether this is purely incidental or whether there might be a more causative relationship [11]. Several mechanisms to potentially explain the association between malignancies and immune-mediated nephropathies have been suggested, however the particular concurrent diagnosis between RCC and AAV may indicate a more complex interplay between the conditions [11]. Interestingly, in this case the patient was diagnosed with metastatic RCC and subsequently developed kidney dysfunction with haemoproteinuria after commencement of tyrosine kinase inhibitor therapy. Investigation revealed evidence of PR3 positivity and a clinical phenotype in keeping with AAV. This raises the question of whether the presence of RCC or its immunomodulatory treatment created an environment that predisposed to the development of AAV.

## Case report

A 53-year-old male presented with a background history of clear cell RCC having undergone a left nephrectomy in 2010. There was no evidence of nodal, lymphatic, or vascular invasion intra-operatively or on imaging post-nephrectomy. He completed five years of follow up surveillance without disease recurrence. He was otherwise well with no other medical or family history of note.

**Fig. 1:**
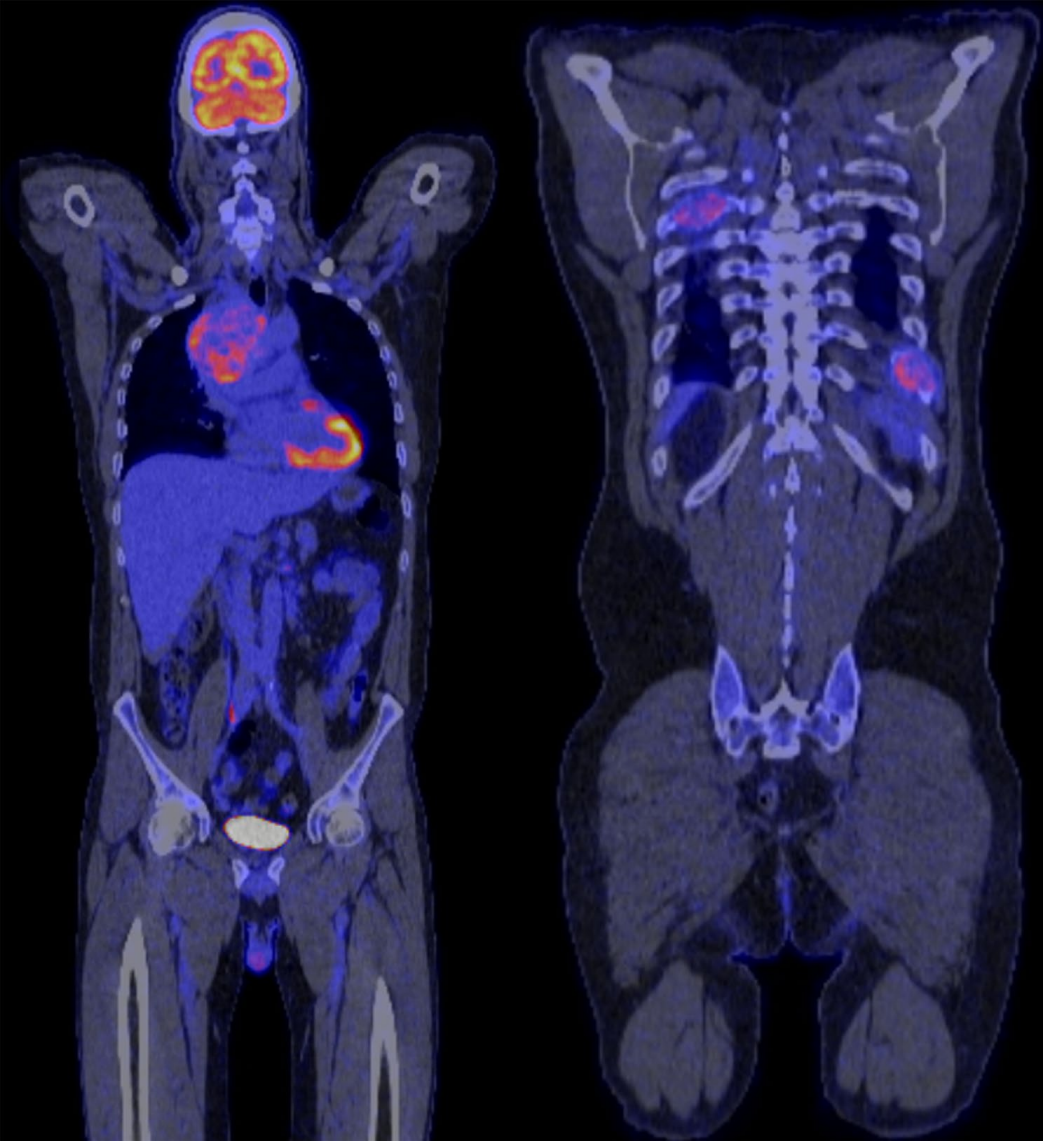
PET scan from time of diagnosis of RCC metastatic disease

Ten years post nephrectomy he consulted an Ear, Nose and Throat surgeon for sinus congestion. There were no concerning features on nasal endoscopy, and management consisted of sinus washes and mupirocin ointment. Three months later he presented to his General Practitioner with a persistent cough and was found to have a large right hilar mass compressing the lower bronchus, with extensive mediastinal lymphadenopathy and multiple foci of bony rib metastases. The left renal bed and right kidney were unremarkable on CT (computed tomography) scan. His serum creatinine at presentation was 102 μmol/L and CKD-EPI eGFR 75 mL/min, with no haematuria. A PET (positron emission tomograpy) scan revealed a necrotic mass in the right hilum with avidity of the subcarinal and para-aortic nodes adjacent to the left renal bed (Fig. 1). There was evidence of widespread metastases throughout the ribs and sacrum. A biopsy of the parabronchial nodes revealed metastatic RCC.

He was commenced on sunitinib and by day 7 of cycle 1 he noticed increasing tongue swelling and sunitinib was ceased. By day 11 of cycle 1 he had increasing dysphagia and mouth ulcers as well as a low-grade fever and sinus congestion. He was admitted to hospital and received several days of intravenous (IV) antibiotics. Dexamethasone was commenced at 8 mg twice daily per oral (PO) to treat mucositis. Chest CT revealed that the hilar mass was causing superior vena cava obstruction, which was treated with 20 Gy over five fractions of palliative radiotherapy. During this admission there was one episode of epistaxis, mild haemoptysis and a patchy rash to the hands and feet. His kidney function remained at baseline and there was no evidence of haematuria. Sunitinib was ceased due to severe mucositis.

After 2 weeks, a trial of Pazopanib was commenced and oral dexamethasone was weaned to 4 mg twice daily. By day 14, cycle 1 of Pazopanib he was admitted to hospital with low grade fevers and fatigue. He was noted to have an acute kidney injury with his SCr 124 μmol/L (baseline 80–100 μmol/L) which was presumed secondary to dehydration and IV fluids were prescribed.

**Fig. 2:**
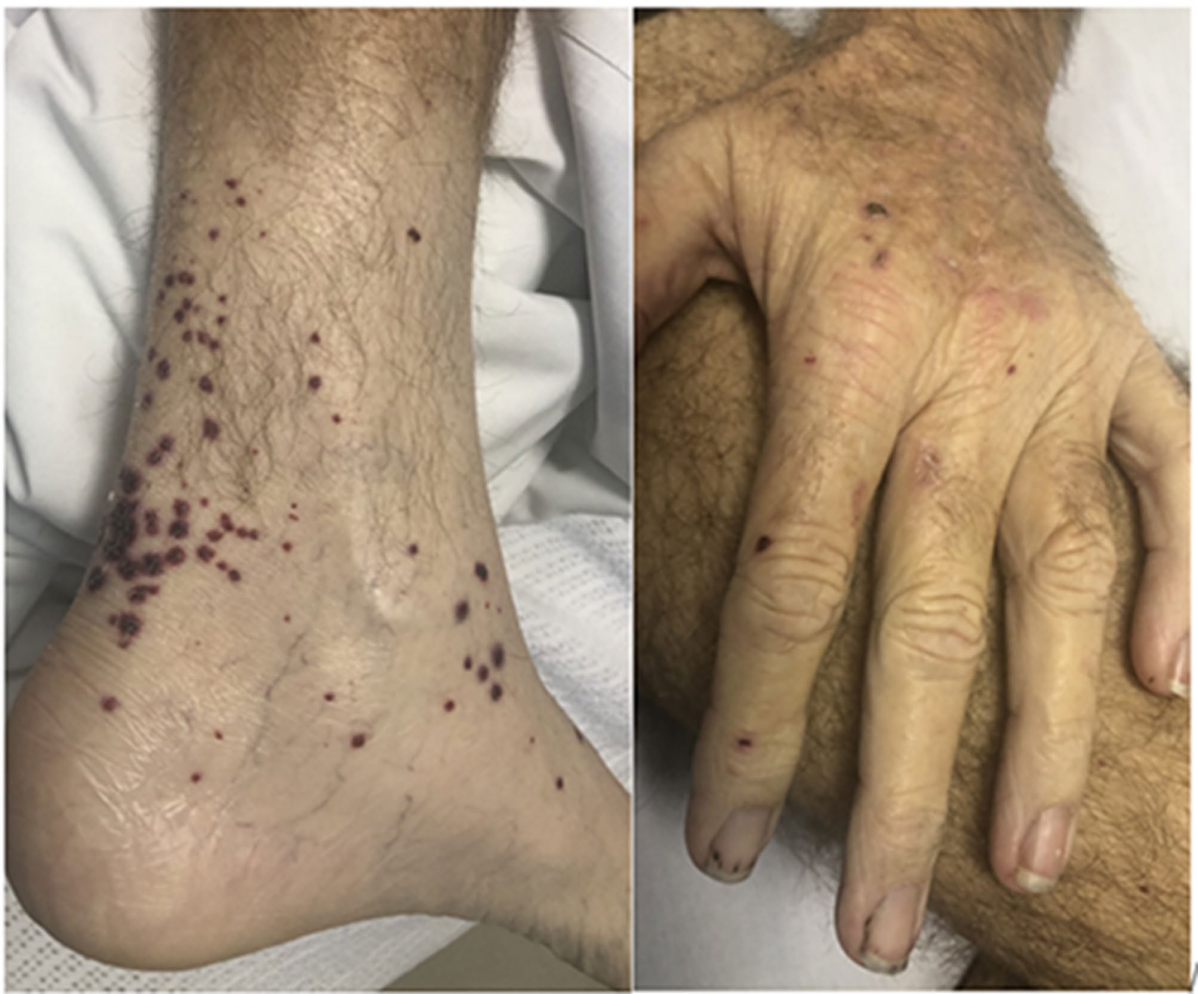
Vasculitic Rash

The patient complained of a new rash on his lower limbs and hands. Over the subsequent three days his SCr rose to 268 μmol/L. Urine microscopy showed microscopic haematuria with > 500 erythrocytes and 40 leucocytes with urine protein:creatinine ratio (PCR) 117 mg/mmol [1040 mg/g]. On examination he was euvolaemic with no infective symptoms. He had a painful, non-blanching purpuric rash on the ankles, feet, thighs, and elbows (Fig. 2). Blood cultures were completed and remained negative. An echocardiogram was also unremarkable with no valvular abnormalities or vegetation.

Kidney ultrasound was unremarkable with expected hypertrophy of his right kidney to 15.3 cm bipolar length. Broader glomerulonephritis screen revealed a negative ANA, ENA, anti-GBM, negative viral hepatitis screen and negative myeloma screen and ESR 89. A vasculitic screen revealed a positive c-ANCA > 2560 and PR3 > 1000 CU. C3 was raised at 2.34 g/L, with normal C4; whilst a low C3 has been associated with poorer prognosis in AAV, studies have shown that elevated C3 can be associated with RCC malignancy [12]. In the setting of a purpuric rash, sinus congestion, rapid deterioration of kidney function with haemoproteinuria, and a strongly positive PR3 titre a diagnosis of Granulomatosis with polyangiitis was made. It was now suspected that the sinusitis he suffered five months prior may have been a prodromal phase of AAV. Other differentials considered at this stage included endocarditis associated vasculitis which was excluded on the basis of absent fevers, negative blood culture and normal echocardiogram. It was considered that the patient’s presentation may have been related to an adverse event from the tyrosine kinase inhibitors. However, the kidney dysfunction at presentation progressed rapidly and developed within a week of Pazopanib commencement which would be an unusual presentation for interstitial nephritis. Given his clinical phenotype at presentation along with the high PR3 level it was felt this was in keeping with AAV.

He received three days of IV 100 mg methylprednisolone and SCr improved to 198 μmol/L. He had ongoing small volume haemoptysis which had been present prior to immunotherapy commencement. A high-resolution CT chest revealed no radiological evidence of AAV pulmonary involvement and therefore plasma exchange was not undertaken. The pre-existing hemoptysis was likely due to the right necrotic hilar metastasis. We did not proceed to kidney biopsy as the patient had clear clinical and serological findings of AAV, as well as the added complexity of solitary kidney status after his previous nephrectomy.

Once IV methylprednisolone was completed, Rituximab was commenced with an induction dose of 2 g IV in a divided dose, one fortnight apart [13–15]. The decision for Rituximab based induction therapy was made in order to reduce the risk of propagation of malignancy, lack of pulmonary involvement at presentation, a serum creatinine that remained under 4 mg/dL and superior remission rates for PR3-ANCA positive patients with Rituximab induction based regimens [16–18]. At time of discharge SCr had improved to 154 μmol/L and urine PCR was 30 mg/mmol (urine ACR was 10 mg/mmol). He continued on 50 mg oral prednisolone for two weeks accompanied by standard prophylaxis including pantoprazole, cholecalciferol and trimethoprim with sulfamethoxazole.

At follow-up appointment two weeks post diagnosis 2.5 mg PO mane ramipril was commenced, the induction rituximab was completed and a steroid weaning plan instituted to reduce by 10 mg/week PO until reaching 10 mg PO daily. At this time, SCr was 132 μmol/L with urine PCR 50 mg/mmol and 30 erythrocytes on urine microscopy. PR3 had fallen to 574 CU.

Approximately two months after diagnosis of AAV he was re-trialed on a tyrosine kinase inhibitor, Axitinib. Kidney function improved to a SCr of 110 μmol/L, close to historic baseline, with no evidence of haematuria or proteinuria. The PR3-ANCA titre improved from > 1000CU at diagnosis to 68CU. Lymphocyte subsets revealed appropriately suppressed CD19 lymphocytes of < 0.02×10^9^/L post-rituximab. Prednisolone had been weaned to a 5 mg PO mane maintenance dose.

**Graph 1:**
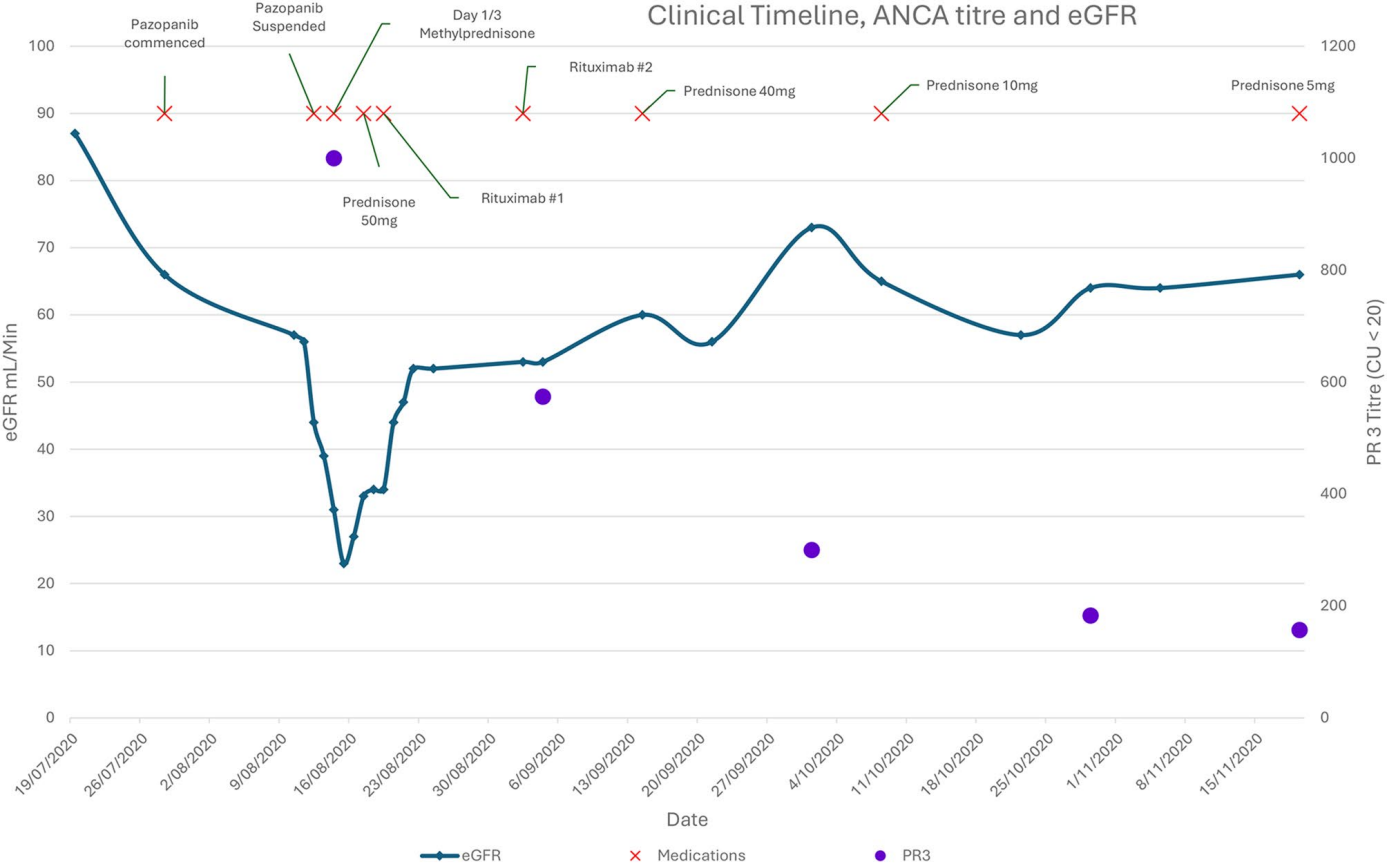
Timeline of clinical events with biochemical markers at initial diagnosis

**Fig. 3:**
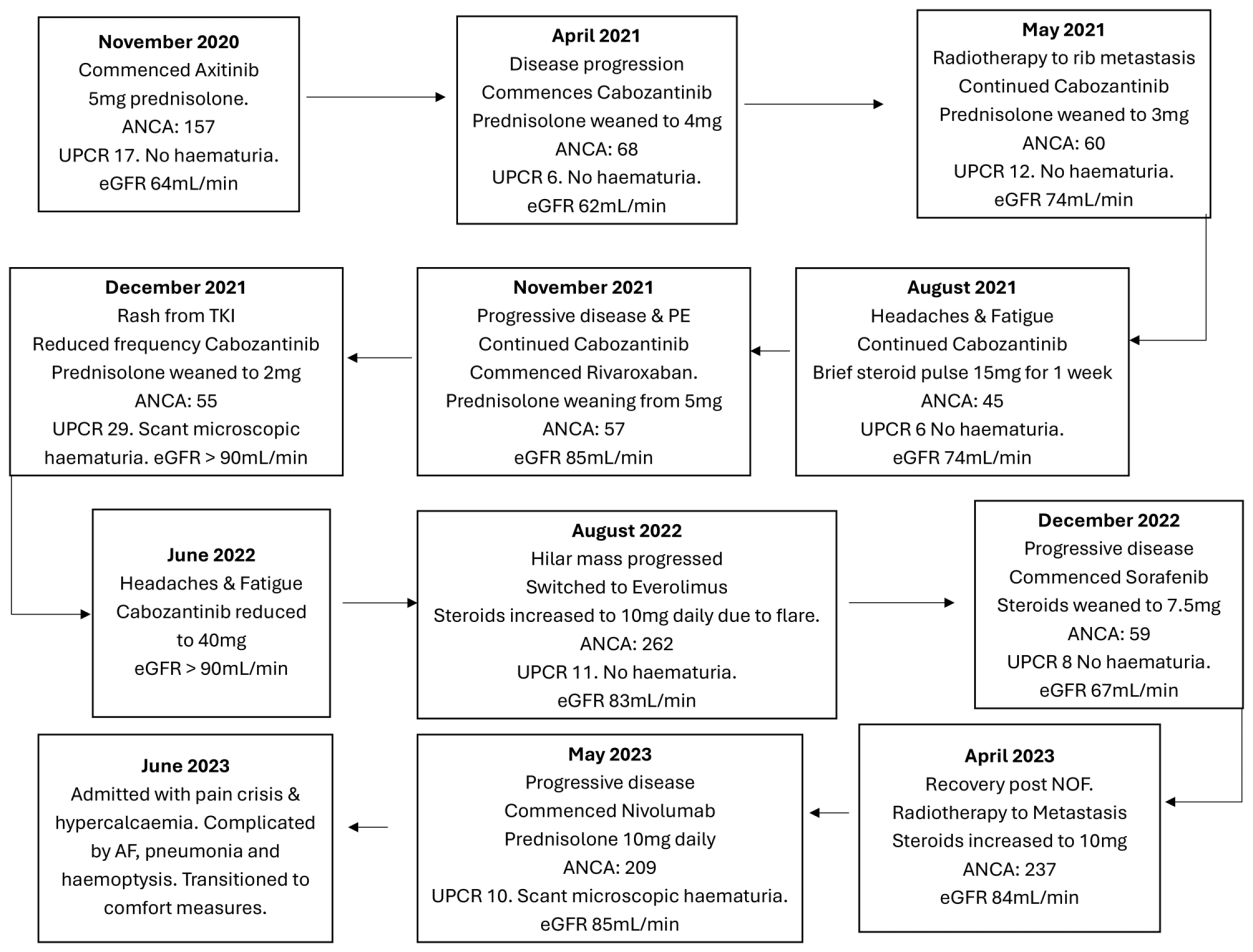
Timeline of oncological management

Over the following 2 years Mr SH underwent a series of treatments for his RCC, a flow chart of this is presented in Fig. 3. A restaging scan revealed progressive disease five months after commencing Axitinib; due to progression, he was switched from Axitinib to Cabozantinib 60 mg daily and further efforts to wean steroids were undertaken. Prednisolone was weaned to 2 mg daily between April and August 2021. Further Radiotherapy was completed to the left posterolateral ribs for pain relief. Due to symptoms of fatigue, headache and Cabozantinib associated rash the prednisolone was increased to 3 mg daily in August 2021, there was no suspicion of AAV recurrence at this stage.

After six months of Cabozantinib therapy a staging scan revealed a pulmonary embolism along with progressive disease. Cabozantinib was continued and prednisolone was weaned back to 2 mg daily and rivaroxaban commenced. Between December 2021 and June 2022 the patient experienced ongoing side effects with rash, fatigue and headaches leading to a reduction in the dose of Cabozantinib to 40 mg daily and a week off of therapy every month.

In August 2022 there was evidence of progressive disease at the hilar mass and decision to cease Cabozantinib and commence Everolimus was made. In addition, the PR3 titre had risen to 262CU along with sinus congestion, this was treated with an increase in steroids to 10 mg daily. PR3 levels then down trended to 59 CU and prednisolone was reduced to 7.5 mg after 1 month.

After 4 months of Everolimus treatment there was progressive disease on CT and therapy was switched to Sorafenib in December 2022, further follow up was complicated by a pathological neck of femur fracture. In April 2023 monitoring of ANCA PR3 titres revealed another increase to 237CU without symptoms. This was treated with increase in prednisolone to 10 mg daily. In the following two months there was progressive disease on imaging and a final line of therapy with Nivolumab was trialed. Unfortunately, the patient became unwell with a pain crisis, hypercalcemia and erosion of the hilar mass into the right bronchus. He transitioned to a focus on comfort measures in Jun 2023 and died in hospital with palliative care.

## Discussion

This case represents a unique example of the development of new onset PR3 positive AAV in a patient with metastatic RCC whilst undergoing treatment with tyrosine kinase inhibitors. The literature describes several cases where RCC has been found either after diagnosis with AAV or concurrently when investigating a patient with AAV [10, 11, 19, 20]. This case is unique in that this patient was already receiving treatment for metastatic RCC when he then presented with symptoms of AAV. It is also more common for paraneoplastic ANCA vasculitis to be MPO positive; in contrast to our case [20]. This case highlights two critical areas for discussion:

- The need for a deeper understanding of the complex interplay between AAV and RCC, and
- The complexity of treating autoimmune vasculitis in the setting of active malignancy. In particular this an area lacking guideline directed care [5].

Tatsis et al present one of the earliest associations between AAV and malignancy where they reviewed 477 patients with granulomatosis polyangiitis and 479 patients with rheumatoid arthritis and screened these patients for either past or concurrent malignancy [11]. They identified 7 patients with granulomatosis polyangiitis also had an RCC whilst only a single patient had a history of RCC within the rheumatoid arthritis cohort. There has been previous recognized association between malignancies and vasculitic processes with the most clearly recognized being leukocytoclastic vasculitis and haematological malignancy [20]. With a number of cases now reported with concurrent RCC and AAV this invites the question, is there a pathological interplay between these two conditions?

Several theories have been offered as to the link between these processes including anti-tumour immune responses leading to cross reactivity of vascular endothelium, common genetic susceptibility pathways, immune complex deposition or excessive cytokine release from tumour cells [10, 20, 21]. To formulate an understanding of the link between AAV and RCC the pathophysiology of each condition must be understood.

The pathogenesis in RCC involves inactivation of the tumour suppressor gene, the Von Hippel-Lindau gene (VHL) which leads to an upregulation of HIF1α (hypoxia inedible factor 1 subunit alpha) and constitutive activation of platelet derived growth factor (PDGF) and vascular endothelial growth factor (VEGF) pathways resulting in increased cell survival, angiogenesis and proliferation [22]. In a metastatic RCC there is evidence that IL-6 levels are greatly increased as part of the tumour milieu to promote evasion of the adaptive immune response by arresting dendritic cell processing and leading to increased intracellular STAT3 signaling [23, 24]. Studies into specific biomarkers for RCC have thus far been unsuccessful but multiple studies have shown elevated circulating levels of IL-6 in patients with RCC [23, 25, 26].

The exact pathophysiology of AAV remains under investigation, however current evidence suggests that loss of self-tolerance to MPO or PR3 antigens lead to autoreactive T cells targeting Neutrophil Extracellular Traps (NETs). These are released by neutrophils within the microvasculature. Immune activation against NETs then leads to disease manifestation in AAV [6]. There is a key role for the autoantibodies against MPO and PR3 in the pathogenesis of AAV, as treatments such as plasma exchange and B cell depleting therapies are effective [6]. In addition to this, the alternative complement system has a role in neutrophil activation, with therapies such as Avacopan, the C5a receptor inhibitor, having comparable efficacy to steroids in the treatment of AAV [27]. The process of stimulating, attracting and activating neutrophils to release NET’s in the microvasculature is a key process in AAV and various studies have shown that elevated levels of IL-6 are key to this process and high circulating levels have been detected in AAV [28–32]. Indeed there have been cases where refractory AAV was treated with Tocilizumab leading to remission [31].

Therefore, there is a suggestion that an overlap between these two conditions may involve dysregulation of immune effector cells and high circulating levels of IL-6. It remains unclear as to the direction of this relationship. Whether the pro-inflammatory state created by AAV with high circulating cytokines and a dysregulation of T regulatory cells leads to a pro-tumorigenic environment for RCC or whether the autocrine activity of an RCC tumour milieu with elevated levels of IL-6 leads to neutrophil priming and pre-disposes to a loss of self-tolerance. Further research and inquiry is required to elucidate the complex connection between these two conditions.

This case also highlights the difficult balance of treating an aggressive autoimmune condition such as AAV in the setting of a patient with metastatic cancer. There is a lack of safety data on the use of immune check point inhibitors in patients with autoimmune diseases as they are excluded from clinical trials due to concern over higher risk of toxicity and deterioration of their auto-immune disease [33]. There is significant evidence to suggest a correlation between the pathophysiological processes underpinning immune related adverse events from checkpoint inhibitors and inherent auto-immune diseases [33–35]. Checkpoint inhibitors continue to gain popularity with increasing evidence including a 2019 study showing Pembrolizumab to have superior effects when combined with Axitinib in RCC compared to sunitinib alone with a 31% lower risk of disease progression[36]. In our case, our patient progressed through several lines of single agent therapy and the use of combined therapy was avoided due to the significant risk of a severe recurrence of AAV and permanent loss of kidney function further limiting his treatment options.

With regards to our patients treatment for AAV he had an excellent response to rituximab based induction therapy. His case of GPA would have ordinarily been treated with maintenance rituximab for a period or azathioprine given high risk of relapse disease [37]. However, in discussion with the patient’s Oncologist we altered standard treatment to maintenance prednisolone and a ‘watch and wait’ approach given the risk of immunosuppressant medication contributing to cancer progression and reduced efficacy of his immunotherapy.

There is increasing use of immune checkpoint inhibitors for solid organ cancers and therefore the utilization of these drugs in patients with active autoimmune diseases will need to be defined. With respect to immunosuppressive treatment of auto-immune conditions it is clear in several reviews that this can reduce the efficacy of checkpoint inhibitor therapy for malignancy treatment and limits treatment options [33, 34]. Further studies in these complex patient cohorts may lead to better understanding of safe therapies that allow for concurrent treatment of both auto-immune disorders and malignancies such as novel steroid sparing agents like Avacopan. It will also be essential to scrutinise information on how the continuation of immunosuppressive treatment of autoimmune disorders affects the treatment of malignancy in those on molecular based targets such as tyrosine kinase inhibitors [38, 39].

The occurrence of AAV in the setting of RCC is increasingly reported within the literature. This case represents a unique timeline in which treatment for metastatic RCC had commenced before there was clinical evidence of an underlying AAV. The main limitation in this case was a lack of confirmatory kidney biopsy in the setting of his single kidney status. There remains a clear but poorly understood connection between these two conditions and the interplay is complex. Indeed in our case the main aim and goal for our patient was to continue to access immunotherapy against his metastatic RCC in order to prolong survival, it was essential that his AAV treatment was developed with this in mind. Further research to understand the pathophysiology of AAV and alternative treatment options will be essential in guiding treatment for patients with concurrent malignancies requiring immunomodulatory therapy in the future.

## Data Availability

Not applicable – This is a case report of a single patient. There is no data set applicable for this report. Clinical Trial number not applicable.

## Abbreviations

AAV: ANCA associated vasculitis
RCC: Renal cell carcinoma
GPA: Granulomatosis with polyangiitis
ANCA: Anti-neutrophil cytoplasmic antibodies
MPO: Myeloperoxidase
PR3: Proteinase 3
CT: Computed tomography
PET: Positron emission tomograpy

## References

[1] Kitching AR, Anders HJ, Basu N, Brouwer E, Gordon J, Jayne DR, et al. ANCA-associated vasculitis. Nat Rev Dis Primers. 2020;6(1):71. 32855422 10.1038/s41572-020-0204-y

[2] J JC, F RJ, F J, F J, T M, J RJ. Comprehensive Clinical Nephrology 6. china: Elsevier:2019.

[3] Yates M, Watts R. ANCA-associated vasculitis. Clin Med (Lond). 2017;17(1):60–64. 28148583 10.7861/clinmedicine.17-1-60 PMC6297586

[4] Kronbichler A, Bond M, Dejaco C. Classification criteria for ANCA-associated vasculitis: One size does not fit all! Rheumatol (Oxford). 2023;62(3):993–95. 10.1093/rheumatology/keac423 35904552

[5] Hellmich B, Sanchez-Alamo B, Schirmer JH, Berti A, Blockmans D, Cid MC, et al. EULAR recommendations for the management of ANCA-associated vasculitis: 2022 update. Annals of the Rheumatic Diseases. 2024;83(1):30–47. 36927642 10.1136/ard-2022-223764

[6] Hutton HL, Holdsworth SR, Kitching AR. ANCA-associated vasculitis: Pathogenesis, models, and preclinical testing. Semin Nephrol. 2017;37(5):418–35. 28863790 10.1016/j.semnephrol.2017.05.016

[7] Guchelaar NAD, Waling MM, Adhin AA, van Daele PLA, Schreurs MWJ, Rombach SM. The value of anti-neutrophil cytoplasmic antibodies (ANCA) testing for the diagnosis of ANCA-associated vasculitis, a systematic review and meta-analysis. Autoimmun Rev. 2021;20(1):102716. 33197574 10.1016/j.autrev.2020.102716

[8] Kidney cancer in Australia statistics 2022 [updated 03/01/22. Available from: https://www.canceraustralia.gov.au/cancer-types/kidney-cancer/statistics.

[9] Day D, Kanjanapan Y, Kwan E, Yip D, Lawrentschuk N, Andrews M, et al. Patterns of care for metastatic renal cell carcinoma in Australia. BJU Int. 2015;116.(Suppl3):36–41. 26204961 10.1111/bju.13176

[10] Norris JH, Leeds J, Jeffrey RF. P-ANCA positive renal vasculitis in association with renal cell carcinoma and prolonged hydralazine therapy. Ren Fail. 2003;25(2):311–14. 12739838 10.1081/jdi-120018732

[11] Tatsis E, Reinhold-Keller E, Steindorf K, Feller AC, Gross WL. Wegener’s granulomatosis associated with renal cell carcinoma. Arthritis Rheum. 1999;42(4):751–56. 10211890 10.1002/1529-0131(199904)42:4<751::AID-ANR19>3.0.CO;2-D

[12] Dong Y, Ma Wm, Yang W, Hao L, Zhang Sq, Fang K, et al. Identification of C3 and FN1 as potential biomarkers associated with progression and prognosis for clear cell renal cell carcinoma. BMC Cancer. 2021;21(1):1135. 34688260 10.1186/s12885-021-08818-0 PMC8539775

[13] Bénard V, Farhat C, Zarandi-Nowroozi M, Durand M, Charles P, Puéchal X, et al. Comparison of two rituximab induction regimens for antineutrophil cytoplasm antibody–associated vasculitis: Systematic review and meta-analysis. Acror. 2021;3(7):484–94. 10.1002/acr2.11274 PMC8280814 34114739

[14] Elfishawi M. Journal club review of “comparison of two rituximab induction regimens for antineutrophil cytoplasm antibody-associated vasculitis: Systematic review and meta-analysis”. ACR Open Rheumatol. 2022;4(5):406–09. 35119219 10.1002/acr2.11409 PMC9096519

[15] The pharmaceuticals benefit scheme - rituximab [Available from: https://www.pbs.gov.au/medicine/item/13101M-13095F.

[16] KDIGO 2021. Clinical practice guideline for the management of glomerular diseases. Guideline. 2021. 10.1016/j.kint.2021.05.021 34556256

[17] Unizony S, Villarreal M, Miloslavsky EM, Lu N, Merkel PA, Spiera R, et al. Clinical outcomes of treatment of anti-neutrophil cytoplasmic antibody (ANCA)-associated vasculitis based on ANCA type. Ann Rheum Dis. 2016;75(6):1166–69. 26621483 10.1136/annrheumdis-2015-208073 PMC4908815

[18] Jones RB, Furuta S, Tervaert JW, Hauser T, Luqmani R, Morgan MD, et al. Rituximab versus cyclophosphamide in ANCA-associated renal vasculitis: 2-year results of a randomised trial. Ann Rheum Dis. 2015;74(6):1178–82. 25739829 10.1136/annrheumdis-2014-206404

[19] Lloyd M, de Verteuil J, Andrews PA. Renal vasculitis associated with renal cell carcinoma. J R Soc Med. 2002;95(6):305–06. 12042383 10.1258/jrsm.95.6.305 PMC1279918

[20] Pankhurst T, Savage CO, Gordon C, Harper L. Malignancy is increased in ANCA-associated vasculitis. Rheumatology (Oxford). 2004;43(12):1532–35. 15316126 10.1093/rheumatology/keh374

[21] Tsimafeyeu I, Leonenko V, Kuznetsov V, Semenkova E, Bondarenko A, Demidov L. Paraneoplastic vasculitis in patients with metastatic renal cell carcinoma. Cancer Rep (Hoboken). 2019;2(2). 10.1002/cnr2.1142 PMC7941514 32721112

[22] Webber K, Cooper A, Kleiven H, Yip D, Goldstein D. Management of metastatic renal cell carcinoma in the era of targeted therapies. Intern Med J. 2011;41(8):594–605. 21627746 10.1111/j.1445-5994.2011.02540.x

[23] Kumar A, Kumari N, Gupta V, Prasad R. Renal Cell Carcinoma: Molecular aspects. Indian J Clin Biochem. 2018;33(3):246–54. 30072823 10.1007/s12291-017-0713-y PMC6052717

[24] Graves A, Hessamodini H, Wong G, Lim WH. Metastatic renal cell carcinoma: Update on epidemiology, genetics, and therapeutic modalities. Immunotargets Ther. 2013;2:73–90. 27471690 10.2147/ITT.S31426 PMC4928369

[25] Miki S, Iwano M, Miki Y, Yamamoto M, Tang B, Yokokawa K, et al. Interleukin-6 (IL-6) functions as an in vitro autocrine growth factor in renal cell carcinomas. FEBS Letters. 1989;250(2):607–10. 2787758 10.1016/0014-5793(89)80805-1

[26] Pastore AL, Palleschi G, Silvestri L, Moschese D, Ricci S, Petrozza V, et al. Serum and urine biomarkers for human renal cell carcinoma. Dis Markers. 2015;2015:251403. 25922552 10.1155/2015/251403 PMC4398943

[27] Jayne DRW, Merkel PA, Schall TJ, Bekker P. Avacopan for the treatment of ANCA-associated vasculitis. N Engl J Med. 2021;384(7):599–609. 33596356 10.1056/NEJMoa2023386

[28] Abdulahad WH, Lamprecht P, Kallenberg CG. T-helper cells as new players in ANCA-associated vasculitides. Arthritis Res Ther. 2011;13(4):236. 21888687 10.1186/ar3362 PMC3239339

[29] Hong Y, Eleftheriou D, Hussain AA, Price-Kuehne FE, Savage CO, Jayne D, et al. Anti-neutrophil cytoplasmic antibodies stimulate release of neutrophil microparticles. J Am Soc Nephrol. 2012;23(1):49–62. 22052057 10.1681/ASN.2011030298 PMC3269928

[30] Berti A, Warner R, Johnson K, Cornec D, Schroeder DR, Kabat BF, et al. The association of serum interleukin-6 levels with clinical outcomes in antineutrophil cytoplasmic antibody-associated vasculitis. J Autoimmun. 2019;105:102302. 31320177 10.1016/j.jaut.2019.07.001 PMC7217333

[31] Nakazawa D, Masuda S, Tomaru U, Ishizu A. Pathogenesis and therapeutic interventions for ANCA-associated vasculitis. Nat Rev Rheumatol. 2019;15(2):91–101. 30542206 10.1038/s41584-018-0145-y

[32] Arimura Y, Minoshima S, Kamiya Y, Tanaka U, Nakabayashi K, Kitamoto K, et al. Serum myeloperoxidase and serum cytokines in anti-myeloperoxidase antibody-associated glomerulonephritis. Clinical Nephrology. 1993;40(5):256–64. 8281714

[33] Kennedy LC, Bhatia S, Thompson JA, Grivas P. Preexisting autoimmune disease: Implications for immune checkpoint inhibitor therapy in solid tumors. Journal of the National Comprehensive Cancer Network J Natl Compr Canc Netw. 2019;17(6):750–57. 31200356 10.6004/jnccn.2019.7310

[34] Johnson DB, Beckermann KE, Wang DY. Immune checkpoint inhibitor therapy in patients with autoimmune disease. Oncology (08909091). 2018;32(4):190–94. 29684232

[35] Alpuim Costa D, de Almeida S B, Coelho Barata P, Quintela A, Cabral P, Afonso A, et al. Pazopanib-induced cutaneous leukocytoclastic vasculitis: An exclusion diagnosis of a multidisciplinary approach. Case Rep Oncol. 2017;10(3):1041–49. 29387004 10.1159/000484402 PMC5788070

[36] Rini BI, Plimack ER, Stus V, Gafanov R, Hawkins R, Nosov D, et al. Pembrolizum ab plus axitinib versus sunitinib for advanced renal-cell carcinoma. N Engl J Med. 2019;380(12):1116–27. 30779529 10.1056/NEJMoa1816714

[37] King C, Druce KL, Nightingale P, Kay E, Basu N, Salama AD, et al. Predicting relapse in anti-neutrophil cytoplasmic antibody-associated vasculitis: A systematic review and meta-analysis. Rheumatol Adv Pract. 2021;5(3). 10.1093/rap/rkab018 PMC8407598 34476335

[38] Kwilas AR, Donahue RN, Tsang KY, Hodge JW. Immune consequences of tyrosine kinase inhibitors that synergize with cancer immunotherapy. Cancer Cell. 2015;2(1). 10.14800/ccm.677 PMC4440700 26005708

[39] Ferguson FM, Gray NS. Kinase inhibitors: The road ahead. Nat Rev Drug Discov. 2018;17(5):353–77. 29545548 10.1038/nrd.2018.21

